# Effects of vibrotactile feedback and grasp interface compliance on perception and control of a sensorized myoelectric hand

**DOI:** 10.1371/journal.pone.0210956

**Published:** 2019-01-16

**Authors:** Andres E. Pena, Liliana Rincon-Gonzalez, James J. Abbas, Ranu Jung

**Affiliations:** 1 Department of Biomedical Engineering, Florida International University, Miami, FL, United States of America; 2 School of Biological & Health Systems Engineering, Arizona State University, Tempe, AZ, United States of America; University of Illinois at Urbana-Champaign, UNITED STATES

## Abstract

Current myoelectric prosthetic limbs are limited in their ability to provide direct sensory feedback to users, which increases attentional demands and reliance on visual cues. Vibrotactile sensory substitution (VSS), which can be used to provide sensory feedback in a non-invasive manner has demonstrated some improvement in myoelectric hand control. In this work, we developed and tested two VSS configurations: one with a single burst-rate modulated actuator and another with a spatially distributed array of five coin tactors. We performed a direct comparative assessment of these two VSS configurations with able-bodied subjects to investigate sensory perception, myoelectric control of grasp force and hand aperture with a prosthesis, and the effects of interface compliance. Six subjects completed a sensory perception experiment under a stimulation only paradigm; sixteen subjects completed experiments to compare VSS performance on perception and graded myoelectric control during grasp force and hand aperture tasks; and ten subjects completed experiments to investigate the effect of mechanical compliance of the myoelectric hand on the ability to control grasp force. Results indicated that sensory perception of vibrotactile feedback was not different for the two VSS configurations in the absence of active myoelectric control, but it was better with feedback from the coin tactor array than with the single actuator during myoelectric control of grasp force. Graded myoelectric control of grasp force and hand aperture was better with feedback from the coin tactor array than with the single actuator, and myoelectric control of grasp force was improved with a compliant grasp interface. Further investigations with VSS should focus on the use of coin tactor arrays by subjects with amputation in real-world settings and on improving control of grasp force by increasing the mechanical compliance of the hand.

## Introduction

Current upper-limb prosthetic limbs are limited in their ability to provide direct sensory feedback to users, resulting in increased reliance on visual cues and attentional demands [1,2]. The use of visual feedback for estimating the degree of hand aperture can be unreliable under many conditions, including visual occlusion or limited lighting. Moreover, determining and controlling grasp force outputs from visual feedback alone can prove a more difficult task than aperture control in the absence of visibly noticeable object deformation. This can result in damage to the object being handled (i.e. crushing or dropping the object) even under optimal visual conditions [3,4]. Sensory feedback from prosthetic limbs could enable amputees to better control the prosthesis in a graded manner (i.e. gradually change grasp force and aperture), perform precise tasks while reducing attentional demands [5], and potentially promote prosthesis embodiment [6,7], thereby improving their quality of life.

Some upper-limb body-powered (BP) prosthesis users are able to get indirect feedback in the form of proprioceptive and grasp force cues from their prostheses [1,5]. This results from the mechanical coupling between the user and the prosthesis through the harness. This mechanical feedback is one reason BP prostheses are often preferred by amputees when performing tasks that require precise handling [5]. Recent advances in prostheses controlled by electromyography (EMG) signals (i.e. myoelectric prostheses) offer considerable advantages over BP prostheses, including lower energy expenditure by the user [2], improved appearance, and increased functionality through advanced EMG decoding, multiple grip patterns, and more degrees of freedom [8–10]. However, myoelectric prostheses do not provide sensation comparable to the mechanical feedback provided by BP prostheses, which limits functionality and increases attentional demands [2,5,11], thereby hindering users’ acceptance of the advanced myoelectric prostheses.

In an effort to address this limitation and to improve myoelectric control performance, several approaches to providing direct sensory feedback (i.e. delivering information derived from sensors on the prosthesis directly to the user) have been investigated. These include invasive peripheral nerve electrical stimulation [12–20], non-invasive transcutaneous electrical nerve stimulation [21–23] and non-invasive cutaneous mechanical stimulation [4,6,7,24–40]. Vibrotactile sensory substitution (VSS) is a form of cutaneous mechanical stimulation in which the missing sensory information (e.g. hand aperture) is encoded through an alternate sensory channel by delivering tactile information from a vibrating device (tactor) placed at a specific location on the prosthesis user’s skin [1,4,24,29,33–35,37–39]. VSS has gained popularity as a sensory feedback approach because the tactors are small, inexpensive and unobtrusive [29,34] and the technique has potential to improve control of grasp force and hand aperture without visually attending to the degree of force and aperture resulting from the myoelectric operation of the hand.

The efficacy of a VSS feedback system for upper limb prostheses depends on its ability to reliably deliver discriminable levels of stimulation that can be readily interpreted, i.e. to deliver a set of stimuli that are distinguishable from each other and that convey task-related information such as hand aperture or grasp force. VSS configurations and mapping strategies to encode the sensory feedback information that have been investigated include the use of amplitude, frequency, or burst-width modulated individual actuators, arrays of tactors to spatially-encode information, or a combination [4,6,7,24,29–39,41,42]. Two of the most promising approaches have been burst-width modulation of an individual actuator, which has been shown to provide a suitable impression of varying intensities [29], and sequential stimulation from an array of tactors, which has been shown to be readily interpretable and avoids habituation [40,43].

The feedback delivered by a VSS system should not only be discriminable, but also provide information which could improve myoelectric control. Earlier efforts to characterize the effects of VSS on prosthetic hand control revealed limited evidence of grasp force control improvement when compared to no feedback conditions [4,29]. In addition, VSS feedback of grasp force has been shown to produce significant performance improvements only in a subset of experienced EMG users [29]. This limitation might be due to the fact that myoelectric control of grasp force output is a rather difficult task, even for experienced myoelectric prosthetic hand users [2,5,44]. Poor performance might be due to the lack of articulating fingers, limited degrees of freedom, and/or the low mechanical compliance of the prosthesis [45,46]. The natural human hand is inherently compliant, in part because of the compliance of skin and the mechanical arrangements of muscles, ligaments, joints and fingers. This feature increases our ability to grade force output as we manipulate objects [47–50]. Most commercially available myoelectric prostheses are much less compliant than the biological hand, and this may limit the ability to control force in a graded manner [51–53], thereby hampering the ability to manipulate fragile objects even if direct sensory feedback is provided. That is, the high demands imposed by a rigid interface could mask any potentially benefits provided by augmentative sensory feedback.

This work was directed at investigating the potential benefits of various VSS designs and the effect of grasp interface compliance on the quality of perception and control of grasp force and hand aperture with a Motion Control “ProHand” myoelectric hand instrumented with grasp force and hand aperture sensors (as described in Materials and Methods). We chose to compare two VSS configurations that have been independently studied [4,29,54] but not directly compared: a single burst-rate modulated voice coil actuator (A1) and a spatially-distributed array of five eccentric mass coin tactors (T5). We also compared both configurations using a stiff and a compliant grasp interface, during force control tasks only, to investigate whether increasing the mechanical compliance of the myoelectric hand improves force control. We investigated the performance of these VSS configurations under independent placement and encoding strategies, purposely chosen so that these configurations could be potentially used to deliver different channels of information simultaneously. Characterizing the performance of each approach would facilitate selection of a suitable configuration for delivering information about each feedback variable (grasp force, hand aperture).

We hypothesized that the ability to convey graded levels of sensory information (grasp force and hand aperture) is greater with a tactor array than with a single burst-rate modulated actuator. This was tested by quantifying the ability of the subjects to discriminate various stimulation levels in paradigms that involved vibrotactile stimulation only and vibrotactile stimulation during active myoelectric control of grasp force and hand aperture of a prosthesis. We further hypothesized that graded myoelectric control of grasp force and hand aperture is better in the presence of VSS feedback delivered using a tactor array than with a single burst-rate modulated actuator. This was tested by quantifying the ability of the subjects to accurately reach different target levels using active myoelectric control of grasp force and hand aperture of a prosthesis. Finally, we hypothesized that graded myoelectric control of grasp force output would be improved when there is a compliant interface between the prosthesis and the force sensor. Results from this study could inform the design of sensory feedback-enabled prostheses, and the methodology used in this study could be readily translated to investigations of VSS with amputee subjects.

## Materials and methods

### Subjects

Written informed consent was obtained from 32 subjects in compliance with the Institutional Review Board of Florida International University which approved this study protocol. All prospective subjects were required to complete a screening questionnaire to determine eligibility. Subjects were able-bodied, over 18 years old, with no sensory disorders and no previous experience using a myoelectric hand. The experiments described in this study were completed in three stages, with different participant cohorts for each experiment. Three male and 3 female subjects (first cohort, 24.0±3.5 years old) completed a basic level discrimination experiment for both VSS configurations (see below). Seven male and 9 female subjects (second cohort, 22.1±3.6 years old) completed experiments to compare VSS performance on perception and myoelectric hand control during grasp force and hand aperture tasks. Finally, 7 male and 3 female subjects (third cohort, 26.4±2.8 years old) completed experiments to assess the effect of interface compliance on the control performance of grasp force levels. All subjects were right handed, except for one female in cohort 2.

### Stimulation configurations

**Fig 1:**
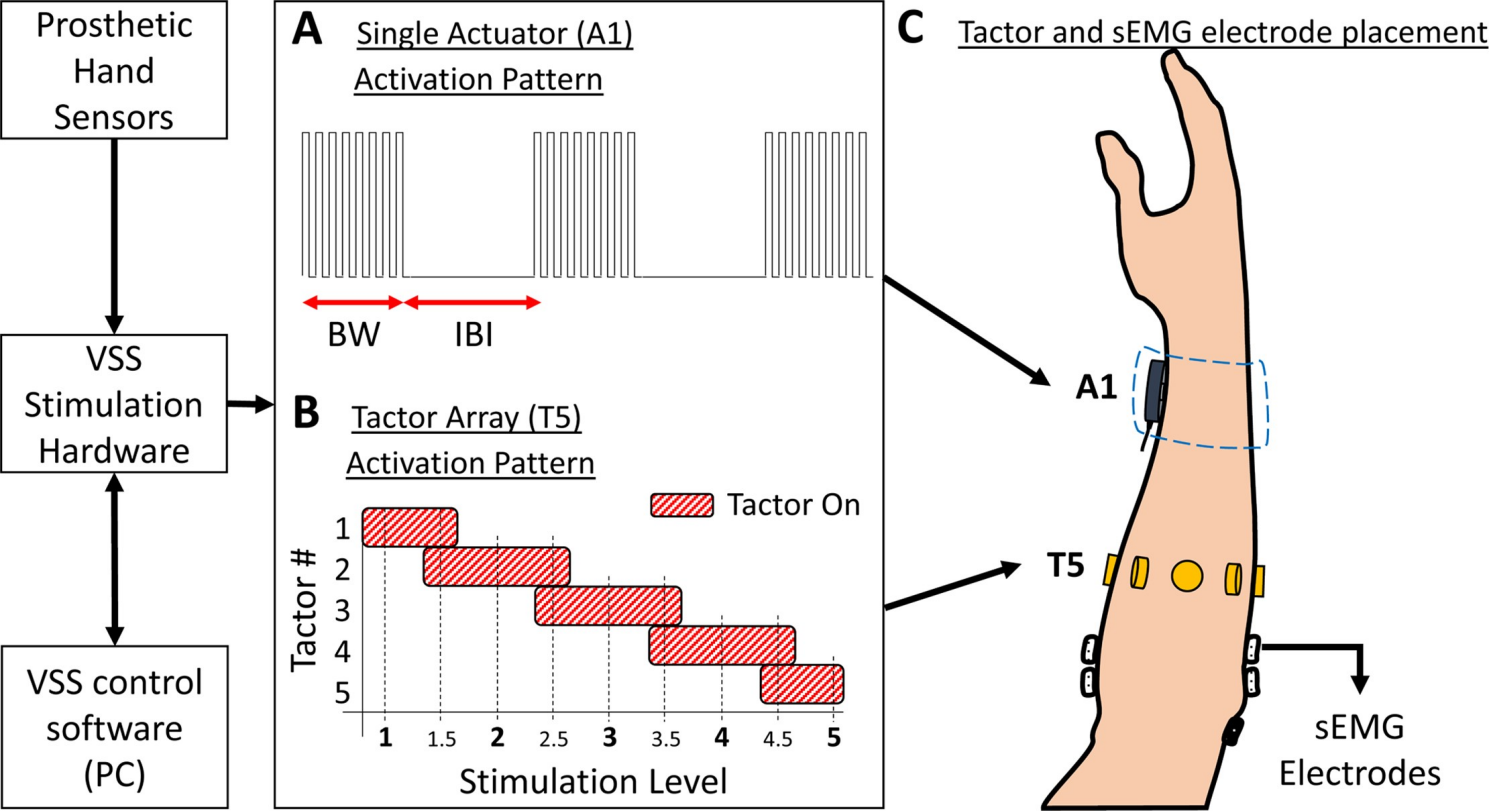
Vibrotactile sensory substitution (VSS) configurations. A) Activation pattern for A1 using bursts of square wave pulses with a fixed pulse frequency of 90Hz, and variable Burst Witdh (BW) and Inter-Burst Interval (IBI). B) Activation pattern for T5 conveying nine distinct stimulation levels. C) A1 was placed on the ventral aspect of the forearm, under a wristband. T5 was placed around the dorsal aspect of the forearm. Surface electromyography (sEMG) electrodes were placed proximal to the elbow.

We developed two VSS configurations (Fig 1) to deliver vibratory stimulation patterns to the forearm based on analog signal readings from Hall-effect sensors that measured grasp force and hand aperture of an off-the-shelf prosthetic hand. The A1 configuration included a single linear actuator (C-2, Engineering Acoustics, Inc., Casselberry, FL, USA) placed on the ventral aspect of the right forearm, about 4 cm from the wrist joint; this location for the single actuator configuration was selected based on prior reports [4] and was confirmed in preliminary tests. This linear actuator (Ø = 30.5mm, L = 7.9mm) has a moving “contactor” (Ø = 7.6mm) that oscillates perpendicular to the skin when an electrical signal is applied. It has a mechanical resonance in the 200–300 Hz range, coinciding with peak sensitivity of the Pacinian and Meissner’s corpuscles which are glabrous skin mechanoreceptors [36,55]. The actuator was activated using bursts of square wave pulses (Fig 1A) with a fixed pulse frequency of 90 Hz, which was selected during pilot testing based on subjects’ reports of stimuli detection quality and comfort at the chosen location. Stimulation intensity levels were increased by decreasing burst width (BW) and inter-burst interval (IBI), thereby resulting in an increase in stimulation burst frequency and duty cycle. Just noticeable difference (JND) tests were performed during pilot studies to determine minimum changes in BW and IBI to convey noticeably different intensity levels. At the lowest stimulation level, BW was 120ms while IBI was 500ms. The maximum stimulation intensity consisted of an IBI of 0ms which meant a constant 90Hz stimulation (no bursts). These stimulation levels were mapped to the prosthetic hand’s full range of grasp force or hand aperture levels.

The T5 configuration included an array of five coin type tactors (Dura Vibe 910–101, Precision Microdrives, London, UK) placed radially on the right forearm (~3 cm average separation [31,34]) no less than 10 cm distal to the elbow joint. This was the most proximal location with the largest forearm circumference that allowed for the desired distance between coin tactors without interfering with myoelectric control. Each coin tactor in the T5 configuration is an eccentric rotating mass (ERM) brushless vibration motor (Ø = 10mm, L = 3mm) driven by a 3 VDC supply with a peak vibration frequency of about 250 Hz. The signal levels in this case were modulated by activating the individual coin tactors in a spatially-distributed manner (Fig 1B). We implemented overlapping activations in between the 5 main signal levels in order to effectively increase the number of signal levels from 5 to 9 discrete, sequential levels. This produced the sensation of the stimulus moving across the arm. These signal levels were mapped to the prosthetic hand’s full range of grasp force or hand aperture levels.

Custom stimulation controller software was developed (LabVIEW, National Instruments, Austin, TX, USA) to map the different signal levels and to control the VSS hardware. All tactors were held in place using double-sided adhesive skin interface tape (SC-F03, Trigno). A stretchable tennis wristband was used to further secure A1 in place. The A1 and T5 configurations were used for all experiments in this study. Note that both configurations used devices that delivered vibrotactile stimulation to the skin and therefore could be described as tactors. We refer to A1 as an actuator and T5 as a tactor to be consistent with the terminologies used by the manufacturers and to emphasize the differences in the mechanisms used to produce the vibrotactile stimuli.

### Experimental setup

**Fig 2:**
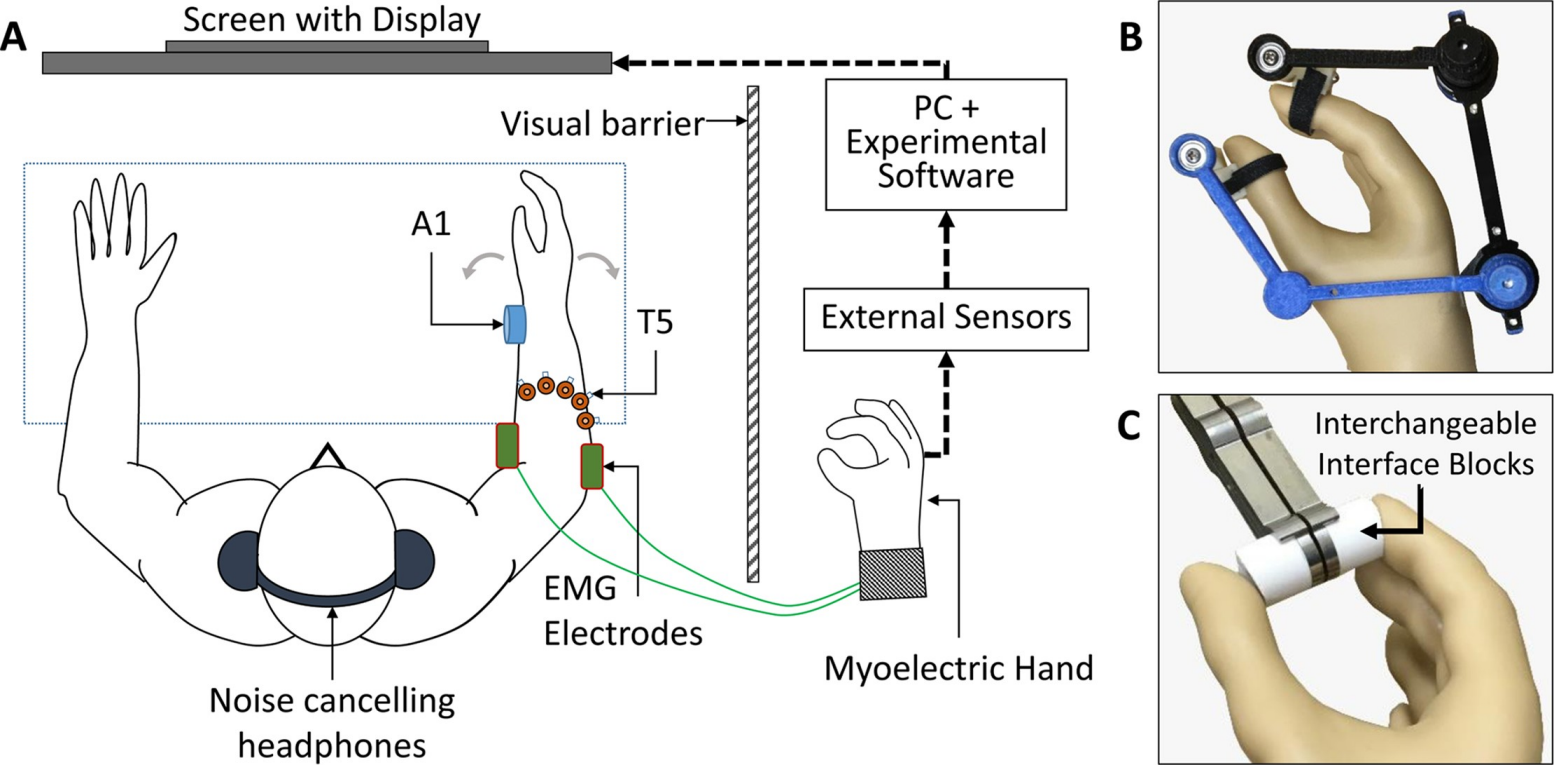
Experimental setup used during all perception and myoelectric control experiments. A) Subjects were seated on a chair and fitted with both VSS configurations, surface EMG electrodes and noise cancelling headphones. A screen in front of the subject displayed visual cues and feedback signals. A sensorized prosthetic hand was placed out of the subject’s field of view, coupled to external grasp force or hand aperture sensors. B) A hand aperture sensor assembly coupled to the thumb and index finger of the prosthetic hand. C) A grasp force sensor assembly with interchangeable interface blocks, placed between the thumb and the index finger of the prosthetic hand.

Subjects were seated on a chair and fitted with both VSS configurations and surface electromyography (sEMG) electrodes. They were instructed to keep both arms resting on a table in front of them, and their right hand’s palmar surface parallel to the vertical plane. A computer screen was located in front of the subject at eye level (Fig 2). The screen displayed visual cues of the grasp force or hand aperture targets and, in trials that used feedback, a visual indicator of performance. An MC “ProHand” instrumented myoelectric prosthetic hand (Motion Control, Inc., Salt Lake City, UT, USA) with built-in grasp force and hand aperture sensors was mounted on a table adjacent to the subject. The prosthetic hand was placed out of the subject’s sight and was coupled to an external hand aperture or grasp force sensor (Fig 2C and 2D). The external force sensor’s grasp interface was either stiff (ABS plastic block) or compliant (PDMS silicone block) (Fig 2C). Custom software was utilized to display the grasp force or hand aperture targets and monitor and record the subject’s responses through the external sensors attached to the prosthetic hand. Subjects were instructed to look forward, face the monitor display at all times, and use their right hand to perform all tasks. If reporting from the subject was required, they were asked to speak clearly, respond as soon as possible and not change their answers. Subjects were fitted with a pair of noise cancelling headphones playing soft white noise to obscure sounds from the stimulator, the prosthetic hand motor, and other sources.

#### System fitting and calibration

Subjects were fitted with a 2-channel EMG system (Motion Control, Salt Lake City, Utah) on the right forearm (Fig 1C). Two pairs of pre-gelled surface EMG electrodes (BlueSensor N-00-S, Ambu, Ballerup, Denmark) were placed distal to the elbow and parallel to the underlying muscle fibers of the extensor carpi radialis (wrist extensor) and palmaris longus (wrist flexor). The reference electrode was placed on the olecranon process. Skin at each electrode site was cleaned using an alcohol pad prior to electrode placement. Two sets of active EMG leads were attached to the surface electrodes and connected to the prosthetic hand. EMG signals were recorded, processed, and used to drive the motor in the prosthetic hand using Motion Control’s clinical EMG control system.

EMG fitting and calibration was performed at the beginning of each experiment. Subjects were asked to flex and extend their right wrist (Fig 2A) in order to open and close the prosthetic hand while the experimenter adjusted the EMG gains and threshold values. Subjects were allowed to see the prosthetic hand moving during the fitting session. Following EMG calibration, sensor readings at the minimum and maximum values for the grasp force and hand aperture sensors (average of three readings each) were used in a two-point calibration procedure to determine sensor offsets and gains.

#### Experimental displays

**Fig 3:**
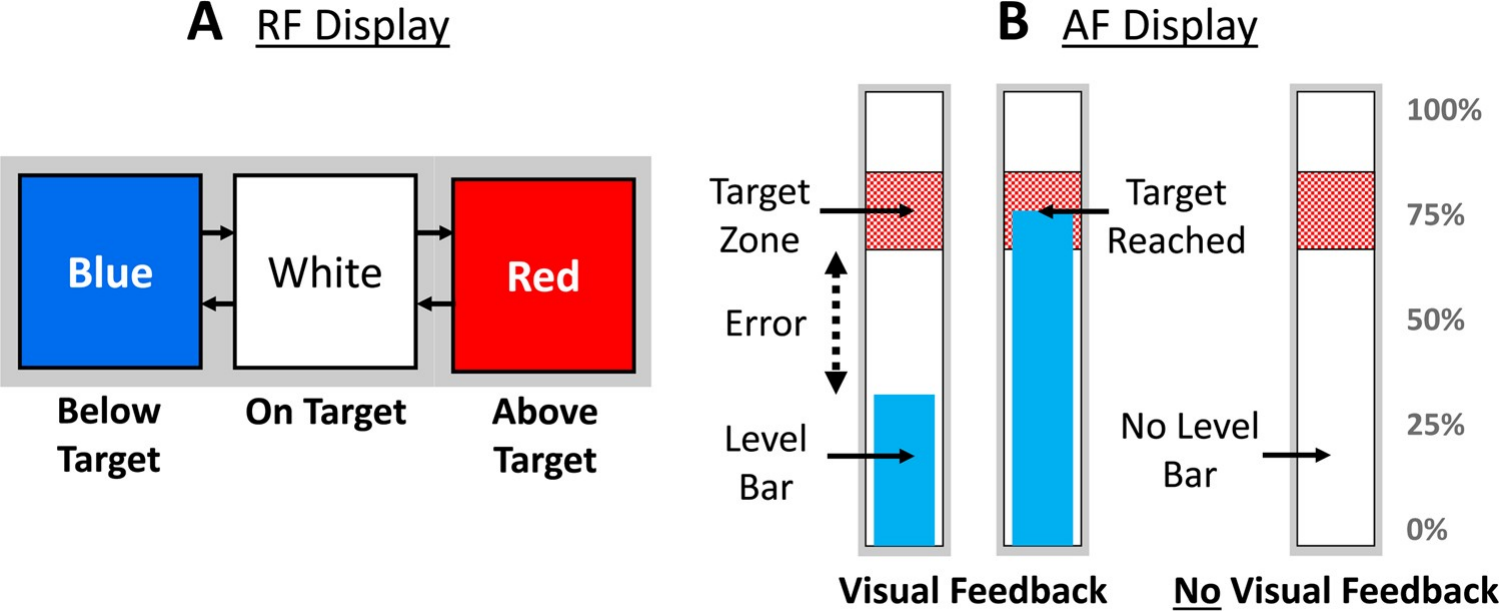
Computer displays for experimental paradigms involving active myoelectric control. A) The relative feedback (RF) display consisted of a colored box showing error information relative to an unknown target. Blue represented below target, red represented above target, white meant the subject was inside the target zone B) The absolute feedback (AF) display consisted of a “thermometer” with a moving level bar (for visual feedback only), and a 10% target zone centered at one of 5 different target levels.

Two different displays were utilized to provide feedback to the subject during trials with active myoelectric control. The relative feedback (RF) display (Fig 3A) consisted of a colored box in which the color represented the error (target level minus actual level) of grasp force or hand aperture. The color encoded the direction of error: blue indicated negative error, red indicated positive error, and white indicated that actual level was within the target zone (10% range). This display was used primarily to avoid giving the subject a visual display of absolute force/position; that is, it enabled the display of a target level without revealing the actual value of the target to the subject). The absolute (AF) display (Fig 3B) consisted of a white thermometer bar scaled to the subject’s calibrated force/hand position range. A moving bar provided absolute feedback of the level of applied grasp force/hand aperture. The moving bar was not visible during the “no visual feedback” condition. A target zone box (width = 10% range) was used to show a target value of 0, 25, 50, 75, or 100% of the total grasp force or hand aperture range.

### Experiments to evaluate perception

The first experimental paradigm was designed to investigate the VSS performance on perception without active myoelectric control (sensory stimulation only). The second paradigm investigated the VSS performance on perception during active myoelectric control of grasp force and hand aperture with a prosthesis. All statistical analyses were performed using Minitab Statistical Software (Minitab Inc., State College, PA) at an alpha level of 0.05 for significance.

#### Perception without myoelectric control

Basic level discrimination experiments were performed to investigate whether subjects could identify five discrete vibrotactile levels (0%, 25%, 50%, 75%, 100%) delivered by A1 and T5 separately to determine the subject’s ability to reliably provide discriminable levels of stimulation. In this paradigm, subjects (cohort 1) were fitted with both VSS configurations as described above. The myoelectric hand was not used in this experiment. The stimulation parameter mapping used for A1 and T5 had been previously determined during pilot experiments. In 3 blocks of 50 non-repeating, randomized stimulation trials each (10 repetitions per level), subjects were instructed to report the level of stimulation perceived. For example, if subjects perceived VSS stimulation at 25%, they would say “25”. The order of the tactor used was alternated across subjects (3 started with A1 and 3 started with T5).

For each trial, the subject’s response was compared to the stimulation level delivered by each VSS configuration. The frequency of correct responses (success rate) during the third block was used as the performance variable for each VSS configuration. Success rates were compared using a paired t-test.

#### Perception during myoelectric control

In this paradigm, subjects (cohort 2) controlled the prosthetic hand to reach a target value of grasp force or hand aperture as shown on the RF display (Fig 3A). Subjects were provided with VSS feedback and a visual display of the error, or the difference between the measured grasp force or hand aperture and the nearest target zone boundary. The quality of the information provided by the VSS configurations about grasp force or hand aperture during myoelectric control was assessed by measuring the subject’s ability to accurately report the target levels after a relative target was reached.

Subjects were instructed to make the display box white by opening and closing (or applying force with) the prosthetic hand while the VSS was active and then hold the position while they reported the level perceived from the VSS feedback. Across 30 trials, 6 repetitions of 5 different levels (0%, 25%, 50%, 75%, 100%) were randomly presented. We compared the subject’s response with the target level in that trial to calculate success rates.

For each trial, the subject’s response (perceived stimulation level) was compared to the value of the presented target. Performance was assessed by analyzing the frequency of correct responses (success rate) and the frequency of responses within a 1-level margin of error. Statistical analysis was performed using a general linear model (GLM) function and post hoc Bonferroni pairwise comparisons to assess the ability of each VSS configuration to convey graded levels of information during grasping force or hand aperture tasks. Success rates for each experimental condition were calculated using responses from all 5 target levels.

### Experiments to evaluate graded myoelectric control

This paradigm (performed by cohort 2) investigated the VSS performance on graded myoelectric control of grasp force and hand aperture outputs. We assessed the subject’s accuracy at reaching a target grasp force or hand aperture, and time to reach the target level. Subjects were instructed to use the prosthetic hand to apply force (or open the hand) to match the target level as shown in the AF display (Fig 3B) and indicate that they had reached it (by saying “there”) under three different feedback conditions: Visual feedback only (V), Tactile feedback only (T) in which only the target zone box was shown but tactile feedback was provided, and Visual and Tactile feedback together (VT). Experiments involving conditions that did not have visual nor tactile feedback were not performed in the interest of keeping experiment durations to a minimum. Performance under these conditions was not expected to be different from chance. Across 30 trials, the target alternated between the 5 different levels presented randomly. The error and task durations for all trials were measured.

The subject’s grasp force or hand aperture was continuously recorded from the external sensors. Performance was documented by calculating the error, or the difference between the measured output (average grasp force, hand aperture from the last 300ms of the trial) and the nearest target zone boundary. If the measured output was inside the target zone, the error was zero. Otherwise, error was defined as the difference between the measured output (from sensor) and the adjacent target zone boundary (target level ± 5%). Task duration was calculated as the difference between the time of target presentation and the time when the participant reported having reached the target.

Measurement of reach error and task duration were extracted for each trial using a custom MATLAB script. Statistical analysis was performed on these performance measures using a GLM function and *post hoc* Bonferroni pairwise comparisons, to evaluate the VSS configuration performance on myoelectric control during grasping force and hand aperture tasks. All statistical analyses were performed using Minitab Statistical Software (Minitab Inc., State College, PA) at an alpha level of 0.05 for significance. Only data from 25, 50 and 75% target level trials were considered in this analysis.

### Experiments to evaluate the effect of interface compliance on perception and control

The effect of interface compliance on grasp force control performance was investigated by performing experiments to evaluate graded myoelectric control (second and third paradigms above) of grasp force with a stiff interface block and with a compliant interface block (Fig 2C). Each VSS configuration (A1, T5) was tested for each interface block (Stiff, Compliant). These experiments were completed by subject cohort 3.

### Experimental sequence for tasks involving myoelectric hand control

**Fig 4:**
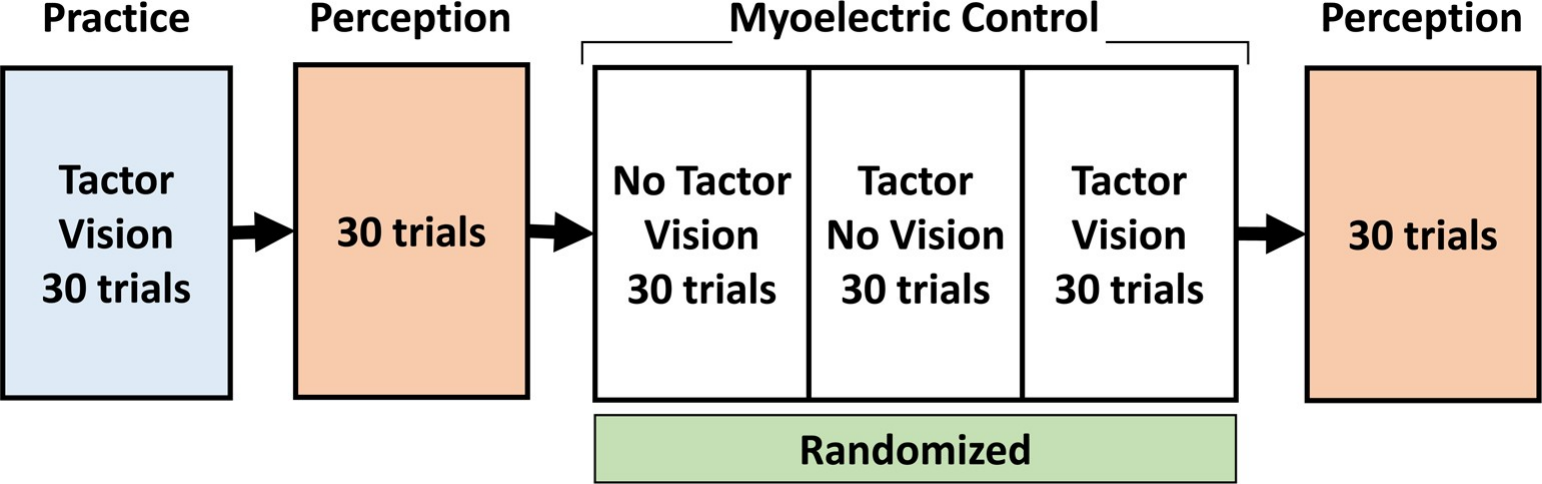
Experimental sequence diagram for experimental paradigms involving active myoelectric control. Subjects first completed a block of practice trials, followed by a block of perception trials. Then, a set of three randomized blocks of myoelectric control trials were performed with variations in visual and VSS feedback. A second block of perception trials was performed to assess training effects on discrimination.

For subjects in cohort 2 and cohort 3, a single experimental sequence (Fig 4) consisted of a block of practice trials, an initial block of perception trials, three randomized blocks of myoelectric control trials under various feedback conditions, and a final block of perception trials. Practice blocks were used at the beginning of every experiment to familiarize the subject with the information provided in the experimental display while receiving visual and tactile feedback together (VT). Subjects were first presented with a sequence of ten VSS stimuli (the five discrete stimulation levels in increasing, then decreasing order) for both VSS configurations to allow them to become familiar with the stimuli. Next, they were allowed to practice myoelectric control while receiving the VSS stimuli for about 2 minutes. Subjects were then given a visual target (using the AF display) and instructed to use EMG control to open or close the prosthetic hand to a certain degree (for hand aperture tasks) or to deliver a certain degree of grasp force to match the target level. In all practice blocks, subjects received both visual and VSS feedback. A set of three randomized myoelectric control blocks were performed in which the presence of visual and tactile feedback was varied. Blocks of perception trials were performed before and after the set of myoelectric control blocks, to test if there were any training effects on discrimination. Analysis focused on the perception blocks and the myoelectric control performance without visual feedback. Table 1 summarizes all experiments performed in this study.

**Table 1:** Summary of experiments performed in this study.

Hypothesis	Subject Cohort	Experimental Task	Feedback Conditions	VSS Configuration	Feedback Variable	Interface Block
Perception	1	Perception-S (sensation only)	T	A1 T5	-	-
	2	Perception-M (during myo control)		A1 T5	grasp force	Stiff
					hand aperture	-
Control	2	Control	T, V, VT	A1 T5	grasp force	Stiff
					hand aperture	-
Interface compliance	3	Perception-M	T	A1^+^ T5^+^	grasp force	Stiff^+^ Compliant^+^
		Control	T, V, VT			

Tasks Perception-S: Evaluating perception with sensory stimulation only; Perception-M: Evaluating perception with sensory stimulation during myoelectric control. Feedback Conditions T: Tactile feedback only; V: Visual feedback only; VT: Visual and Tactile feedback together. VSS Configuration A1: Single actuator; T5: Tactor array

^+^All possible combinations of VSS configuration and interface block used for every task.

## Results

### VSS system performance: Perception

#### Without myoelectric control

Subjects were able to identify discrete VSS stimuli levels from A1 and T5. Success rates during the third block were 79±10.9% and 84±12.7% (mean±STD) for A1 and T5, respectively. No significant performance differences were found between the two VSS configurations.

#### During myoelectric control

**Fig 5:**
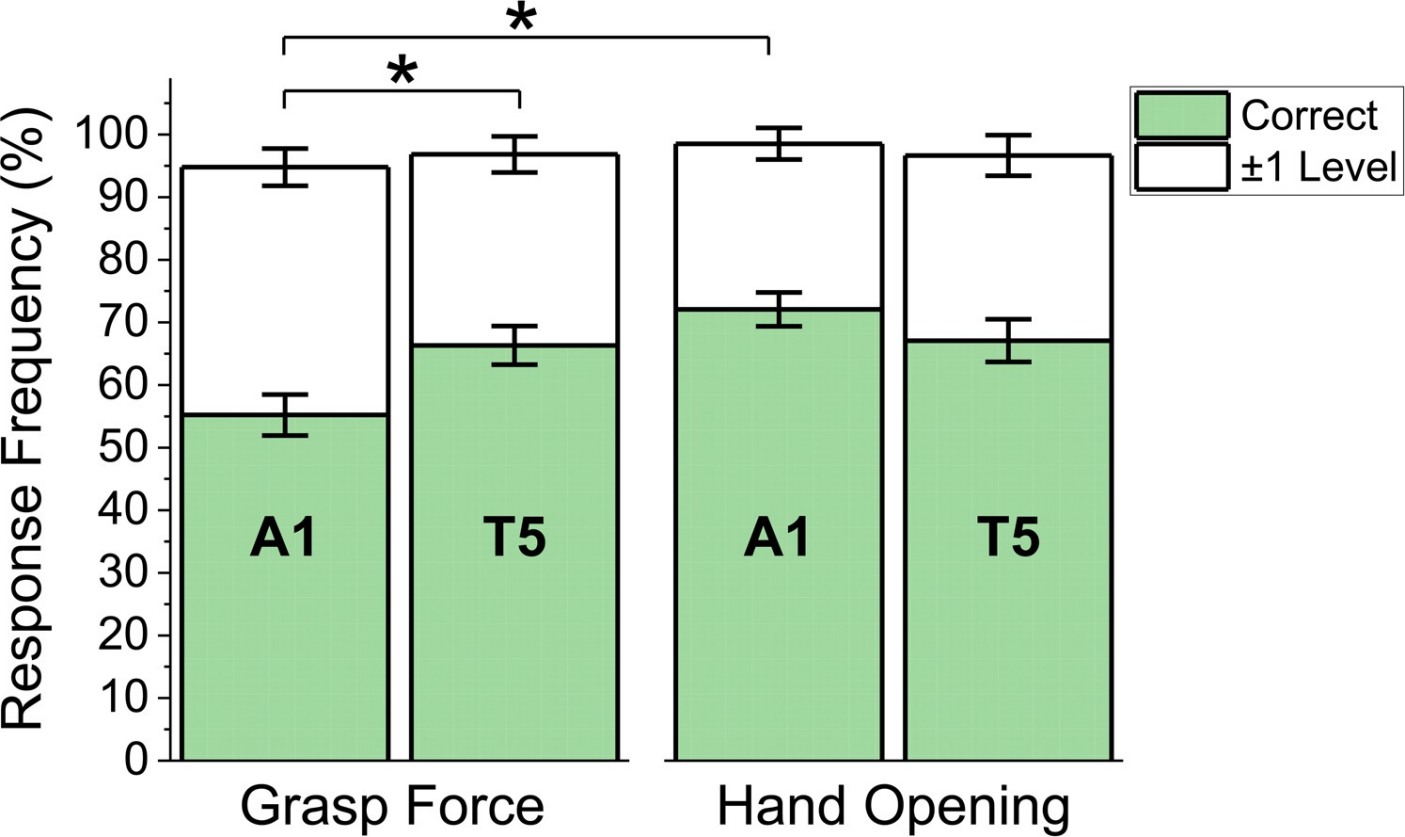
Discrimination performance on perception tasks during grasp force and hand aperture control. Both VSS configurations (A1 and T5) were compared within all subjects (N = 8). Shaded bars: Frequency of correct responses. White bars: Frequency of responses within ±1 level of the actual stimulation level delivered (mean±SEM). * corresponds to significant differences in the frequency of correct responses (*p*<0.05).

All perception results are based on subject response data from all 5 target levels. There were no significant differences between the first and second perception blocks, so they were combined for analysis. Fig 5 shows the frequency of correct responses as well as the frequency of responses within ±1 level of the actual stimulation level delivered (mean±SEM) for each combination of VSS configuration and feedback variable.

Subjects correctly discriminated grasp force levels more frequently (*p* < .05) when using T5 than A1, but there were no significant differences in response accuracy between A1 and T5 for hand aperture tasks. With A1, response accuracy was significantly higher (*p* < .01) during hand aperture tasks than force tasks.

### VSS system performance: Graded myoelectric control

**Fig 6:**
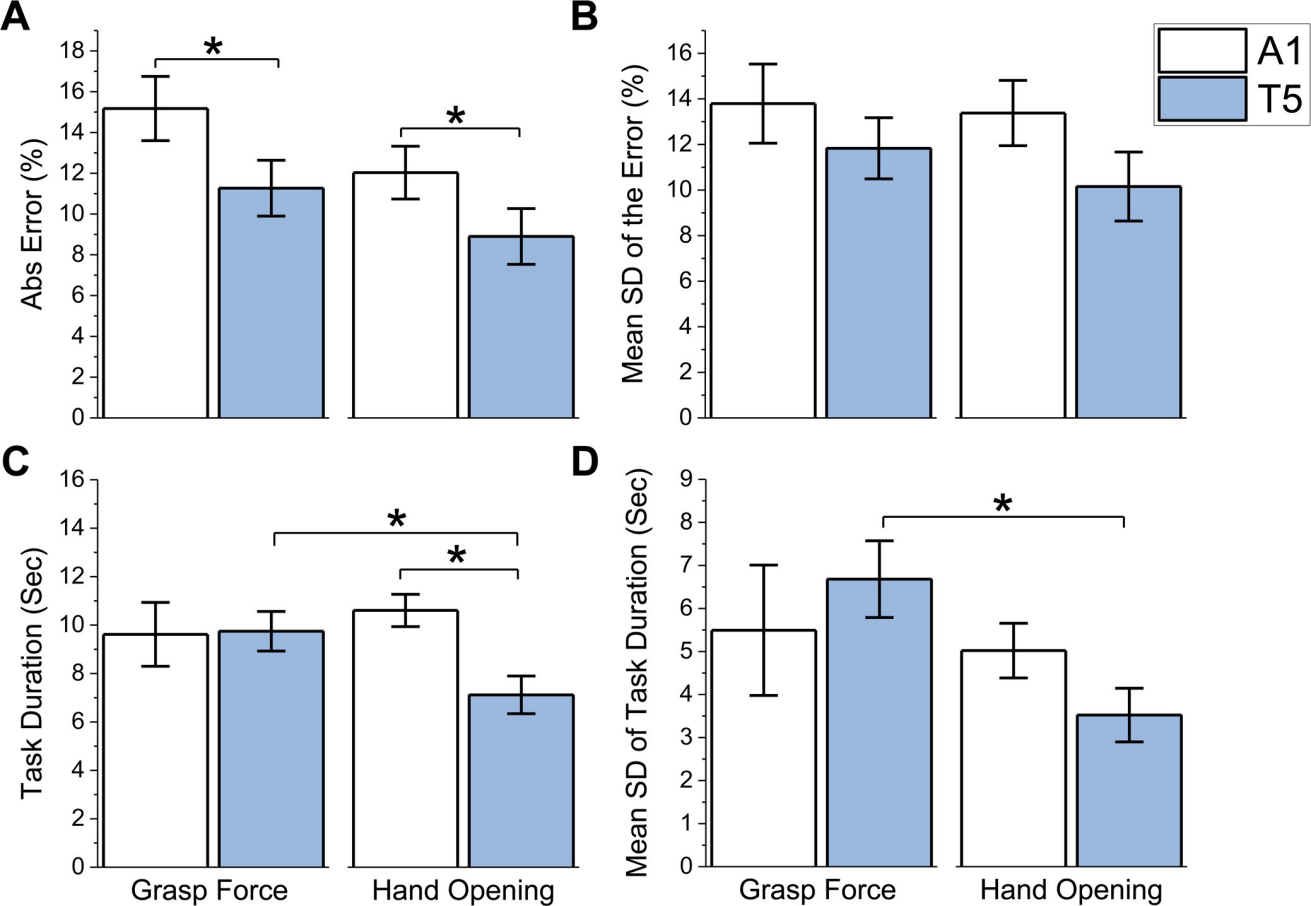
Performance on tasks requiring myoelectric control of grasp force and hand aperture. White bars: A1, Shaded bars: T5 A) Mean absolute value of the reach error B) Variability of the reach error data presented as the mean standard deviation of the reach error C) Mean task duration D) Variability of the task duration data presented as the mean standard deviation of the task duration. All results (N = 8) include their corresponding SEM. * indicates significant difference (*p*<0.05).

No significant performance differences were found between V and VT during graded myoelectric control, suggesting that adding VSS feedback did not negatively affect myoelectric control performance with vision. Fig 6 shows performance results (mean±SEM) for each combination of VSS configuration and feedback variable under T. In the grasp force tasks (Fig 6A—left two columns), subjects performed significantly better when using T5 than A1 (*p* < .01). Similarly, in the hand aperture tasks (Fig 6A—right two columns) subjects performed significantly better when using T5 than A1 (*p* < .05). There was no significant difference in task duration when using A1 or T5 during grasp force tasks (Fig 6C). In contrast, hand aperture tasks took significantly longer to complete (*p* < .001) with A1 than with T5. Additionally, grasp force tasks took significantly longer to complete (*p* < .05) and were significantly more variable (*p* < .05) than hand aperture tasks with T5.

### Effect of interface compliance on perception and control

In this experiment, each subject (N = 10) completed Perception-M (perception during myoelectric control) and Control experimental tasks (Table 1). The variables manipulated were interface block (stiff vs compliant) and VSS configuration (A1 vs T5) for the grasp force feedback variable only.

**Fig 7:**
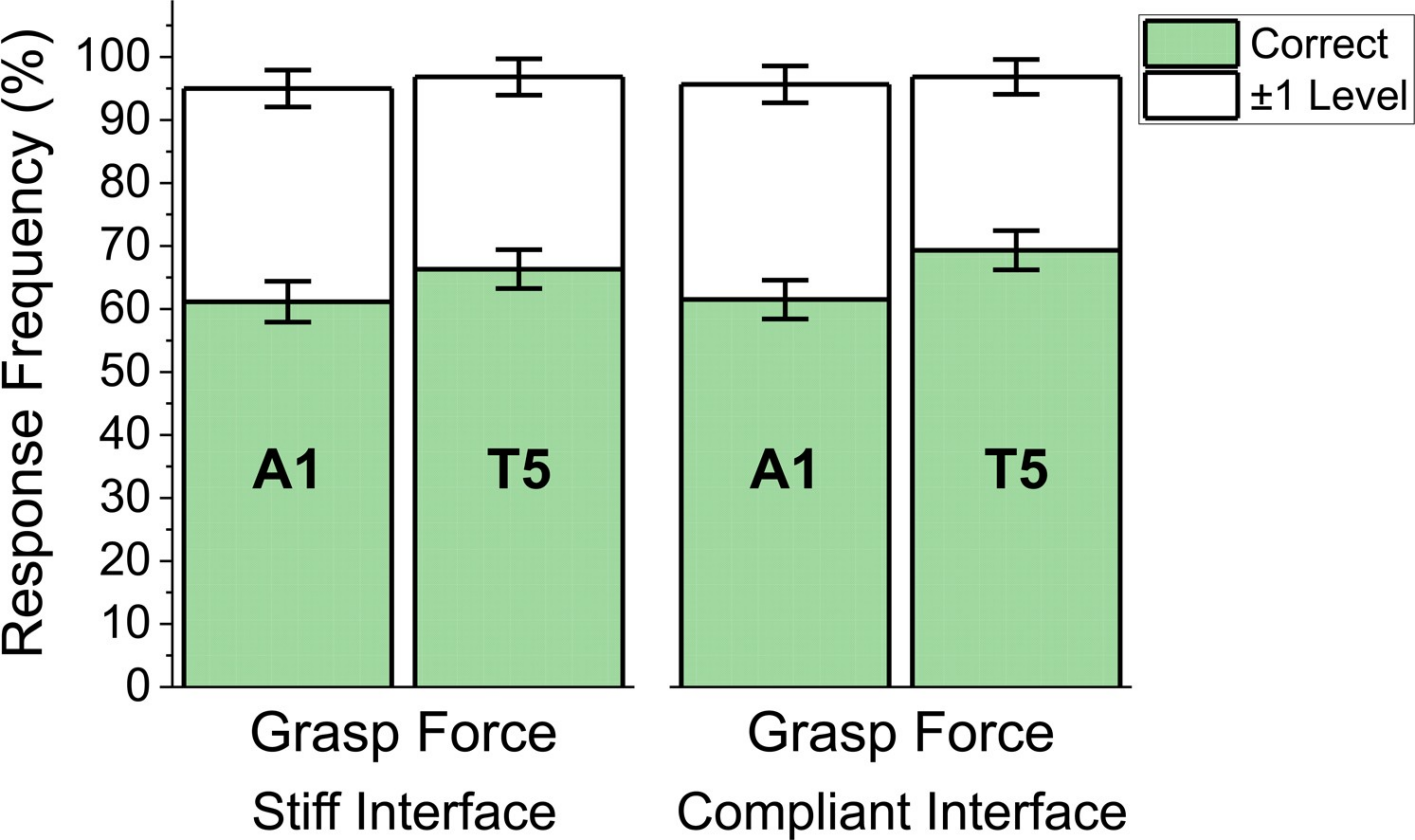
Discrimination performance on perception during grasp force control with stiff and compliant grasp interfaces. Both VSS configurations (A1 and T5) were compared within all subjects (N = 10). Shaded bars: Frequency of correct responses. White bars: Frequency of responses within ±1 level of the actual stimulation level delivered (mean±SEM).

All perception results are based on subject response data from all 5 target levels. Since no significant differences were found between the first and second perception blocks, they were merged for analysis. Fig 7 shows the frequency of correct responses as well as the frequency of responses within ±1 level of the actual stimulation level delivered (mean±SEM) for each combination of VSS configuration and grasp interface block type. There were no significant differences in VSS discrimination accuracy scores across the set of VSS configurations and grasp interface block type.

**Fig 8:**
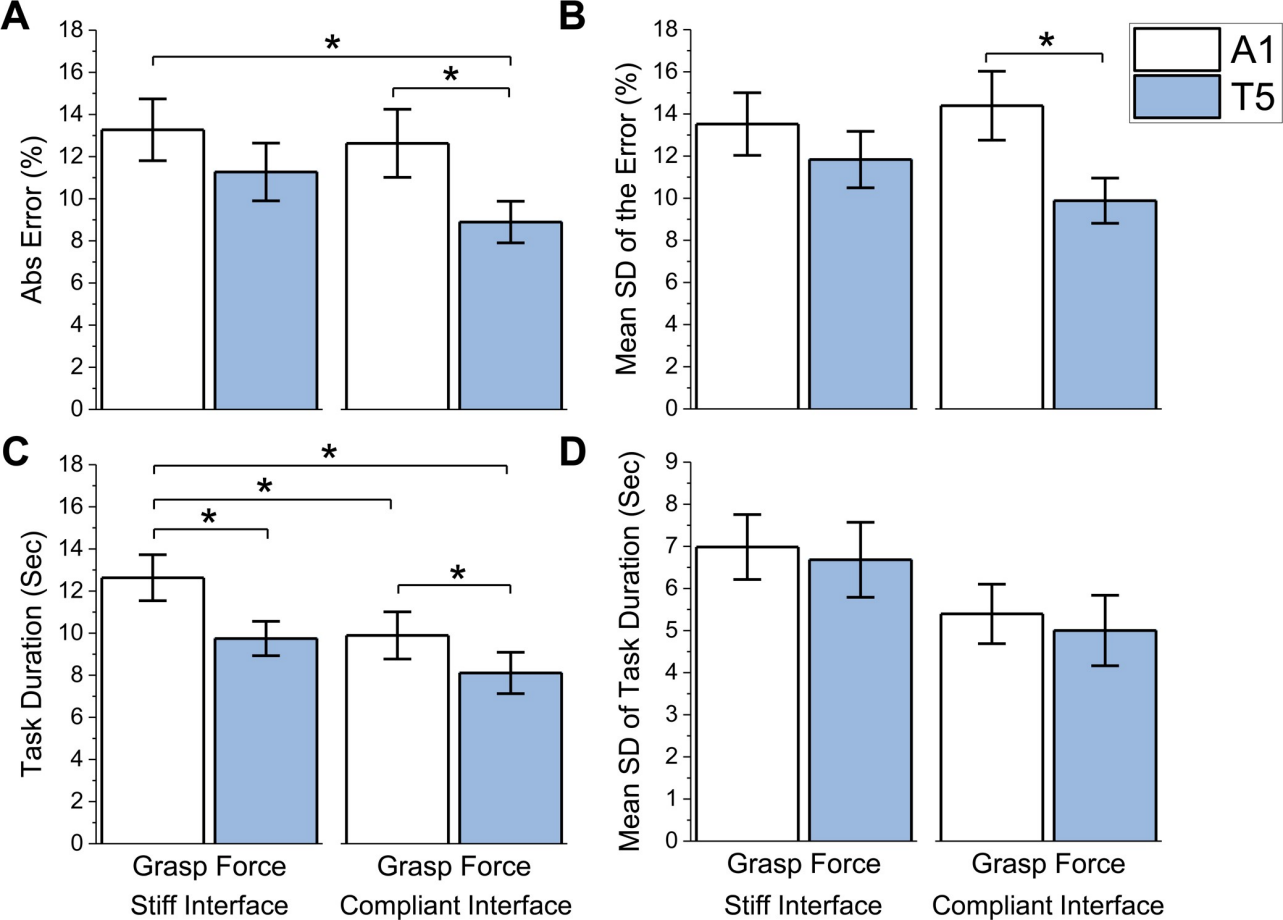
Performance on myoelectric grasp force control tasks with stiff and compliant grasp interfaces. White bars: A1, Shaded bars: T5. A) Mean absolute value of the reach error. B) Variability of the reach error data presented as the mean standard deviation of the reach error. C) Mean task duration D) Variability of the task duration data presented as the mean standard deviation of the task duration. All results (N = 10) include their corresponding SEM. * indicates significant difference (p<0.05).

Similarly, no significant performance differences were found between V and VT during graded myoelectric control of grasp force. Fig 8 shows performance results (mean±SEM) for each combination of VSS configuration and grasp interface block type under T. With the compliant interface, subjects performed better with T5 than with A1 (*p* < .01) and had lower variability (*p* < .05); task durations were also significantly shorter with T5 than with A1 (*p* < .05). With A1, task durations were significantly shorter with the compliant interface than with the stiff interface (*p* < .05).

## Discussion

In this study, we developed a vibrotactile sensory substitution (VSS) system using a single burst-rate modulated actuator (A1), and a spatially activated array of five coin tactors (T5). We performed a comparative assessment of these two VSS configurations with able-bodied subjects to investigate the VSS performance on perception and myoelectric control of grasp force and hand aperture tasks with a prosthesis. We also investigated the impact of interface compliance on the ability to perceive graded vibrotactile information, and control grasp force outputs with the myoelectric hand.

We found no significant differences in the subject’s ability to discriminate between the 5 different target levels delivered by a linear actuator or a tactor array, when tested under a stimulation only paradigm. Both VSS configurations conveyed enough information for subjects to detect differences between the desired intensity levels, with high (>78%) and roughly similar levels of accuracy. These results are consistent with previous studies on the perception performance with these VSS configurations [4], and suggest that their performance may be comparable when the user is not performing a motor task, despite differences in device placement and encoding strategies. However, when the user was controlling a myoelectric hand to regulate grasp force, performance on the perception task was better with feedback from the tactor array than with the actuator. There were no significant differences in perception between the actuator and the tactor array during hand aperture tasks. In addition, when using the actuator, perception performance was significantly lower for the grasp force task than the hand aperture task (Fig 5). These differences could be due to differences in cognitive load between the grasp force and hand aperture tasks. The imposition of a cognitive load can impact motor task performance and has been used to improve the ability of outcome assessment procedures to detect and characterize the impact of motor impairments [56]. This cognitive load more strongly affected the actuator’s performance, therefore suggesting that tracking changes in burst timing during force control might be more demanding than tracking changes in stimuli location [39].

In assessing the impact of the VSS configurations on the ability to control a myoelectric prosthesis, the tactor array improved myoelectric control accuracy (Fig 6A) regardless of the task performed. This is consistent with results from the perception performance experiments, which suggest that changes in stimuli location from the tactor array allows subjects to track changes in force and aperture more efficiently than burst timing from the linear actuator. The tactor array also exhibited lower task durations than the actuator during hand aperture tasks, whereas no significant differences in task durations were found for grasp force tasks. This could be due to the inherent difficulties in myoelectric control of grasp force. There were no differences in performance when visual and VSS feedback were both present, as compared to when only visual feedback was present. This suggests that adding VSS feedback did not negatively affect myoelectric control performance with vision.

Results also indicated that added compliance improved control quality: error rates were lower when receiving feedback from the tactor array and task durations were lower with feedback from the linear actuator. Notably, added compliance did not affect perception performance. These results suggest that added mechanical compliance at the interface between the prosthetic hand and the environment could increase the user’s ability to grade force output without impairing force perception.

### Vibrotactile feedback system

The vibrotactile feedback system developed for this study was designed to deliver vibratory patterns to the forearm based on sensor readings from an off-the-shelf prosthetic hand instrumented with grasp force and hand aperture sensors. Vibrotactile stimuli are typically perceived well, with no obvious adaptation effects [4] and do not seem to need the frequent adjustment of stimulation parameters often required during electrotactile stimulation [43,54]. A common limitation with vibrotactile stimulation is that the strength of vibration may be perceived differently based on the area of skin in contact and mounting pressure [29]; Therefore, conveying signal intensity through changes in amplitude or frequency can be inconsistent [31,33,34]. To circumvent this issue, we chose to adjust the width and separation of the square wave tone bursts (burst-rate modulation) delivered by the linear actuator. The burst frequency of the actuator was fixed at 90 Hz. This frequency was found to produce better, more reliable sensations (based on subject reports during pilot studies) than the 200–300 Hz range typically used for this device [4,29]. One of the possible drawbacks of this stimulation configuration is the potential loss of precise timing information due to rapid changes in grasp force or hand aperture [39]. These changes can be delayed or omitted due to the length of the previous burst period, especially when using long burst durations.

Moreover, the details of the mapping from sensor readings to VSS stimulus pulse parameter selection can affect the efficiency of information delivery. A previous study that used a linear sensor-stimulation map did not find objective performance improvements [42], but a study that used a non-linear map did [38]. This might be due to the non-linearity associated with the perception of stimulus change [36] and/or the activation characteristics of the targeted skin receptors [55]. In this study, based on initial results from pilot experiments, we implemented a non-linear (exponential) mapping scheme to modulate the burst-rate of the actuator.

With the tactor array, signal intensity was modulated by varying the location of the tactors activated. Previous studies have shown that subjects can identify individual tactile stimuli down to a distance of 1.5cm between tactors [31,34]. In this study, the tactors were separated by about 3cm in an attempt to improve discrimination performance. This arrangement limited the space available for the array, and hence the amount of sensory information delivered by the array, which is dependent on the number of tactors used. Overlapping activations in-between the 5 main stimulation levels were used to overcome this limitation and provide subjects with up to 9 discrete levels of stimulation.

### Myoelectric control scheme and hand interface compliance

The Motion Control myoelectric hand used in this study was non-backdrivable and the control scheme locked the hand at the current force/position level in the absence of an EMG signal. The hand used a proprietary proportional velocity control scheme [57], in which the magnitude of the EMG signal determines the velocity of the hand motor. As a result, graded control of force or position output was challenging; subjects reach level often oscillated across the target zone. Furthermore, the low mechanical compliance of the hand caused force output levels to rapidly increase when attempting to reach mid-range force targets, which we suspect exacerbates the loss of precise timing information from the vibrotactile stimuli as described earlier. Alternative designs have been shown to allow for coordination and scaling of grip forces more consistent with behaviors observed using the natural hand; these designs include backdrivable, position-controlled prostheses, integrating mechanical compliance and advanced control features [8,10,52,53,58]. We hypothesized that increasing the mechanical compliance of the hand would help mitigate the loss of precise timing information from the vibrotactile stimuli, thus improving the performance of the stimulation map, allowing subjects to better control mid-range force outputs. The performance results from this study (Fig 8) suggest that designing more compliant myoelectric hands could be beneficial and should be further explored.

### Experimental paradigms

#### Perception during myoelectric control tasks

Previous studies investigated VSS performance on the ability to discriminate between different levels of stimulation [4,7,31], however most did not test perception during graded control tasks. Moreover, some of the studies that involved control tasks employed the use of virtual environments, or non-myoelectric control methods (i.e. joystick, mouse) as opposed to an actual myoelectric prosthesis [4,23,30,39,54,59]. For this study, subjects controlled a sensorized myoelectric hand to perform grasp force and hand aperture tasks. We used the relative feedback (RF) display in order to set grasp force or hand aperture targets without revealing the absolute target level to the subjects. This strategy enabled quantification of the ability of the subjects to discriminate various stimulation levels after a myoelectric control task was completed. Future studies could directly compare VSS feedback discrimination performance with and without myoelectric control to investigate whether increased cognitive loading from myoelectric control tasks affects discrimination performance.

#### VSS performance on graded myoelectric control

VSS feedback has been shown to improve control performance when compared to conditions where no feedback at all is provided (visual or vibrotactile) [54]. We expected similar results would be found if we had tested the myoelectric control performance without any feedback. In an effort to shorten the length of the experimental sessions, we did not perform this comparison. Instead, we used the absolute feedback (AF) display to measure the subject’s control performance with visual feedback, while testing with and without VSS to determine if VSS affected control performance while having visual feedback. We then assessed performance with VSS alone in the absence of visual feedback.

With visual feedback, performance was not affected by the presence of VSS feedback. Although the results are not shown here, subjects reliably reached all targets with virtually no error, which precluded assessment of the ability of VSS feedback to improve control performance (*i*.*e*. a ceiling effect), but our results did demonstrate that the presence of VSS feedback did not degrade performance, which is in agreement with previous studies [60,61].

During this study, all subjects were able to reach minimum and maximum values of grasp force and hand aperture without visual or tactile feedback by using their maximum muscle contractions in a bang-bang control fashion. Furthermore, previous studies have found that mid-level targets are more difficult to distinguish with visual feedback alone [29], hence VSS feedback could prove more beneficial in this range. For these reasons, we focused our myoelectric control performance analysis on data from 25, 50 and 75% target level trials.

### Experimental limitations

One limitation of the experiments in this study was that we did not control the direction of target approach. That is, subjects were allowed to oscillate around the target until they sensed they had reached it using the VSS feedback. This precluded analysis of the effect of approach direction on performance. This also meant that the tasks completed by the subjects were not representative of typical daily life activities, where reaching force and position targets with one attempt is often required. Future studies could use a single attempt method in which subjects are instructed to approach the target from one direction and stop once they feel they have reached it. This would emphasize the importance of avoiding oscillatory movements and encourage the use of a strategy that is more akin to that used in daily tasks. Furthermore, the subjects recruited for this study were novice myoelectric users and participated in one experimental session only. Although significant learning effects in myoelectric control performance were not observed in this study, additional repetitions of the tasks may eventually improve performance as multiple experimental sessions have been shown to reduce error rates and task durations [4,29,39,40]. More experienced myoelectric users experience a lower cognitive load for the experimental tasks and may be able to take greater advantage of the feedback to achieve better control performance [29].

### Implications for future work

This study provides comparative data on the performance of VSS feedback configurations for providing grasp force and hand aperture feedback and comparative data on control of grasp force with a stiff and compliant interface. These results may help to inform the design of the mechanics and sensory stimulation strategies for feedback-enabled prostheses. Future studies should include the simultaneous use of hand aperture and force feedback and study the performance at detecting object stiffness [4].

## Conclusions

We investigated the performance of a single burst-rate modulated actuator and a spatially activated array of five coin tactors for providing feedback of grasp force and hand aperture feedback from a prosthesis. Performance on a perceptual task was not different for these two VSS configurations. However, during graded myoelectric control of grasp force and hand aperture, perception of feedback from the tactor array was better than perception from the single actuator. Graded control of grasp force with myoelectric control could be improved by increasing the compliance of the interface between the prosthesis and grasped object. Future studies should investigate the capability of VSS configurations to enhance the functionality of myoelectric prosthetic hands during daily activities in real-world environments.

## Supporting information

S1 Dataset Vibrotactile sensory substitution study performance data. (ZIP)
